# Contrastive learning enhanced pseudo-labeling for unsupervised domain adaptation in person re-identification

**DOI:** 10.1371/journal.pone.0328131

**Published:** 2025-07-14

**Authors:** Xuemei Bai, Yuqing Zhang, Chenjie Zhang, Zhijun Wang

**Affiliations:** 1 School of Electronic Information Engineering, Changchun University of Science and Technology, Changchun, Jinlin, China; 2 High Performance Computing Center, Changchun Normal University, Changchun, Jinlin, China; Wuhan University of Science and Technology, CHINA

## Abstract

Person re-identification (ReID) technology has many applications in intelligent surveillance and public safety. However, the domain difference between the source and target domains makes the generalization ability of the model extremely challenging. To reduce the dependence on labeled data, Unsupervised Domain Adaptation (UDA) methods have become an effective way to solve this problem. However, the influence of pseudo-label generated noise on model training in existing UDA methods is still significant, resulting in limited model performance on the target domain. For this reason, this paper proposes a contrast learning-based pseudo-label refinement with probabilistic uncertainty in the unsupervised domain, adapted to Person re-identification, aiming to improve the effectiveness of the unsupervised domain adapted to Person re-identification. We first enhance the feature representation of the target domain samples based on the contrast learning technique to improve their discrimination in the feature space, thereby enhancing the cross-domain migration performance of the model. Subsequently, an innovative loss function is proposed to effectively reduce the interference of label noise on the training process by refining the generation process of pseudo-labels, which solves the negative impact of inaccurate pseudo-labels on model training. Through a series of experimental validation, the method experiments on two large-scale public datasets, Market1501 and DukeMTMC, and the Rank-1 accuracy of the proposed method reaches 91.4% and 81.4%, with the mean average precision (mAP) of 79.0% and 67.9%, respectively, which proves that the research in this paper provides a good solution for the Person re-identification task with effective technical support for label noise processing and model generalization capability improvement.

## Introduction

Person re-identification (ReID) is a critical task in intelligent video security systems, aiming to retrieve specific pedestrians across a network of non-overlapping cameras [1]. As an important research area in computer vision, cross-domain ReID focuses on accurately matching pedestrians across different surveillance devices and varied scenes, even in the presence of lighting variations, posture differences, and occlusion. This technology has widespread applications in intelligent surveillance, including pedestrian tracking in shopping malls, underground stations, and other public places. In public security, it helps police quickly locate suspects or find missing persons, thereby improving case detection efficiency. Furthermore, in the development of smart cities, ReID supports traffic management and crowd flow analysis, contributing to the optimization of public space safety layouts. However, due to challenges such as cameras with different viewpoints [2], different resolutions [3], lighting variations [4], posture differences [5], occlusion [6], and heterogeneity [7], person re-identification remains a challenging task.

The core of supervised Person re-identification is to train the model to learn feature representations that distinguish different pedestrian identities through labeled pedestrian ID tags. Typically, the training data contains images of the same pedestrian from multiple cameras, and each image is labeled with a unique pedestrian ID or fine-grained attributes (e.g., clothing color, backpack, etc.), and the model is required to extract features robust to changes in pose, occlusion, and illumination through supervised signals. The primary categories encompass deep metric learning, local feature alignment, and re-ranking techniques. Although current supervised person re-identification methods have demonstrated significant performance, they rely on manual labeling, and building such large-scale and labeled datasets is expensive and time-consuming. Moreover, existing solutions often neglect either crossing cameras (e.g., illumination and resolution differences) or pedestrian misalignments (e.g., viewpoint and pose discrepancies), which easily leads to poor generalization capability when adapted to the new domain [8]. Thus, we focus our research on unsupervised person re-identification.

Unsupervised Person re-identification is a pedestrian recognition technique that does not rely on annotated data and focuses on identity matching by analyzing the features of the image itself. This approach is important in practical applications, as it does not require a large amount of labeled data, reduces data preparation costs, and can be quickly adapted to new scenes or cameras. Current UDA Re-ID methodologies can be broadly categorized into two primary classes. The first encompasses UDA Re-ID algorithms predicated on Generative Adversarial Networks (GANs). These algorithms focus on mitigating the distributional discrepancies between source and target domains through style transfer. The second category comprises UDA Re-ID algorithms that leverage pseudo-labeling techniques. These methods aim to assign labels to the unlabeled target domain dataset using pseudo-labels. Given the inherent noise within these generated pseudo-labels, we propose a contrastive learning-based pseudo-label optimization approach to mitigate this issue and enhance the accuracy and reliability of the pseudo-labels. This approach effectively reduces intra-class feature variance and increases inter-class feature variance, thereby promoting more compact clustering. Our primary contributions are summarized as follows:

1. In this study, we proposed a contrast learning-driven agent task to enhance the quality and performance of pseudo-labels generated by K-means clustering. By introducing a contrast learning mechanism, the method can capture the similarities and differences between samples more effectively, thus achieving more accurate label assignment during the clustering process, enhancing the accuracy of pseudo-labels, and providing more reliable training data for subsequent Person re-identification models.

2. In this study, we proposed a contrastive learning-enhanced clustering framework named CLEPR (Contrastive Learning Enhanced Pseudo-Labeling for Unsupervised Domain Adaptation in Person Re-identification) specifically optimized for the person ReID task. Through the design of a novel loss function, the framework leverages the benefits of contrastive learning to strengthen the discriminative power of feature representations and improve clustering performance. Furthermore, this new loss function not only enhances the model’s ability to discriminate but also accelerates the convergence speed of the model training process, reducing computational resource consumption and demonstrating superior efficiency.

3. Demonstrated significant performance improvement on two large-scale Person re-identification datasets DukeMTMC and Market1501, outperforming many traditional methods and achieving excellent results, especially in complex scenes and large-scale data.

## Related work

### Unsupervised domain adaptive methods

Researchers have proposed unsupervised domain adaptation (UDA) methods. Current UDA Re-ID approaches can be broadly categorized into two groups. The first category comprises UDA Re-ID algorithms based on Generative Adversarial Networks (GANs). These algorithms aim to align the source and target domain data through style transfer, thereby mitigating the distribution discrepancy between the source and target domains. The second category includes UDA Re-ID algorithms based on pseudo-labeling. These algorithms focus on assigning labels to the unlabeled target domain dataset using pseudo-labeling techniques. Due to the inherent noise in the generated pseudo-labels, researchers in this area primarily address the challenges of pseudo-label generation and the reduction of pseudo-label noise.

#### Generative adversarial network-based methods.

The core concept behind UDA Re-ID methods based on Generative Adversarial Networks (GANs) involves transferring labeled images from the source domain to the target domain. This is achieved by generating images that emulate the style of the target domain, followed by utilizing the style-transferred images and their associated labels for supervised learning in the target domain, thereby aligning the source and target domains. Zhan *et al*. [9] introduced a spatial-aware UDA Re-ID network capable of adapting to images at both spatial and pixel levels. Tang *et al*. [10] employed GANs in their research, proposing an iterative self-supervised domain adaptation network. Zhou *et al*. proposed a multi-camera translation GAN to transform source dataset images into the multi-camera styles of the target dataset. Deng *et al*. [11] proposed a GAN that preserves self-similarity to maintain domain self-similarity and dissimilarity, learning a mapping function between the two domains. While GAN-based methods effectively address issues such as insufficient data diversity, inter-domain discrepancies, and camera style variations in person re-identification tasks, the limited regional and temporal scope of existing person re-identification datasets, particularly the lack of sufficient cross-view pedestrian data, especially concerning pose and appearance variations, hinders the model’s ability to learn discriminative or invariant features. Furthermore, pedestrian images generated using GANs may contain noise due to factors such as illumination, resolution, and camera viewpoint, thus failing to eliminate domain discrepancies between the training and testing sets, which in turn affects the model’s robustness.

#### Pseudo-labeling-based methods.

UDA methods based on pseudo-labeling primarily investigate the inter-class discrepancies within the target domain. Early models addressing UDA problems via pseudo-labeling employed a single-branch pipeline, leveraging self-supervision to learn from target data. Features generated by pre-trained models are utilized to predict pseudo-labels for unlabeled samples, which subsequently facilitate model training. In the context of unsupervised domain adaptation for person re-identification (ReID), pseudo-labeling involves employing a model trained on labeled source domain data to predict and label the unlabeled target domain data. This approach effectively addresses the challenge of unlabeled data and enables the training of supervised models in conjunction with labeled data. Algorithms such as DBSCAN and K-Means are commonly employed for generating pseudo-labels based on clustering.

The generation of pseudo-labels inevitably introduces noise, leading to a discrepancy between the generated labels and the ground truth labels of their corresponding instances. Mitigating the noise within pseudo-labels can enhance the predictive capabilities of the model. Pseudo-label refinement, based on representation learning, initiates with feature extraction from the unlabeled target domain. Subsequently, pseudo-labels are derived through clustering, and reliable samples are identified via algorithmic design, facilitating model fine-tuning. Leveraging the refined model for feature extraction, some researchers have proposed sampling strategies to filter out unreliable samples for reliable sample selection. Fan *et al*. [12] introduced the Progressive Unsupervised Learning (PUL) method. Ge *et al*. [13] proposed the MMT unsupervised mutual mean-teaching network framework, which provides robust soft pseudo-labels through online peer teaching, concurrently training two identical networks. This approach effectively optimizes pseudo-labels by jointly supervising the offline optimization of hard pseudo-labels (with one hundred percent confidence) and the online refinement of soft pseudo-labels (with confidence less than one hundred percent), thereby enhancing the neural network’s performance.

### Contrast learning

Contrast learning is a self-supervised learning method that has achieved significant success in many computer vision tasks. The core idea is to optimize the model by constructing pairs of positive and negative samples so that samples of the same class (positive sample pairs) are as close as possible in the feature space and samples of different classes (negative sample pairs) are as far away as possible. This approach can help the model learn a more discriminative feature representation in the absence of labeled data [14]. Contrast learning has become a recent hotspot in unsupervised learning research, achieving excellent performance in different tasks [15, 16]. The most straightforward strategy for contrastive learning involves the use of data augmentation to construct sample pairs [17]. Specifically, a positive sample pair consists of two augmented views of the same object, while the rest constitute a negative sample pair. Some of the more classic methods are SimCLR [18], MoCo [19], BYOL [20], SwAV [21] and SimSiam [22].

**Fig 1:**
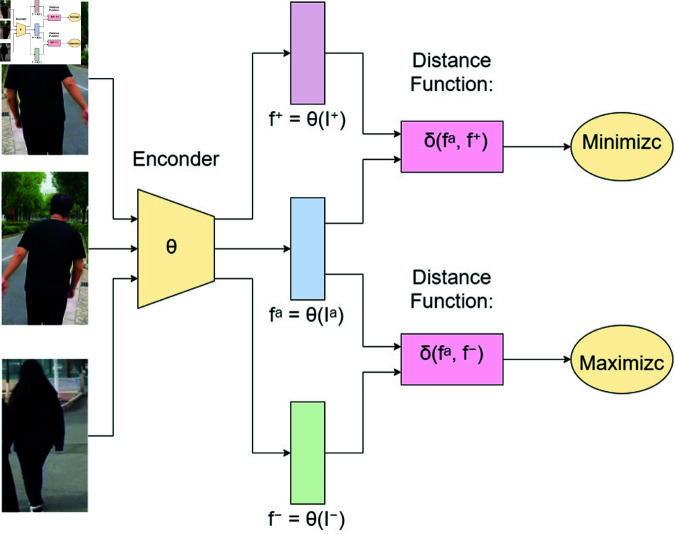
Contrast learning.

Positive sample pairs typically refer to distinct samples belonging to the same class. In the context of Person Re-Identification (ReID), two augmented views of the same pedestrian’s image constitute a positive sample pair. Conversely, negative sample pairs generally represent samples from different classes. In Person Re-Identification (ReID), images of pedestrians with different identities form negative sample pairs. By minimizing the distance between positive sample pairs and maximizing the distance between negative sample pairs, the model can learn discriminative feature representations.As shown in Fig 1.

This paper introduces contrastive learning to cross-domain person re-identification (ReID) to mitigate noise in pseudo-label generation within the target domain. By employing contrastive learning, the model leverages contrastive loss to enhance the discrimination between positive and negative sample pairs, thereby improving the accuracy of pseudo-label generation for target domain images. Optimizing the quality of pseudo-labels, the distinctiveness of each pedestrian’s features in the target domain is amplified. Consequently, pseudo-label generation can better capture subtle differences in pedestrian identities, thus avoiding the risk of misclassification in pseudo-labeling.

## Methodological design

This paper aims to reduce the pseudo-label noise, to make the clustering better and thus assign pseudo-labels more accurately by boosting the features, and to map the images from the original domain to the feature domain with the same pedestrian as small as possible and different pedestrians as large as possible.

In cross-domain Person re-identification, the labeled source domain dataset $S={\left\{(s_{i},y_{i})\right\}}_{i=1}^{N_{s}}$ and the unlabeled target domain dataset $T={\left\{t_{i}\right\}}_{i=1}^{N_{T}}$, the source domain is the one containing *N*_*S*_ labeled pedestrian images, the label associated with the pedestrian images *s*_*i*_ is denoted as *y*_*i*_, the target domain is the one containing *N*_*T*_ unlabeled pedestrian images, *t*_*i*_ denotes their pedestrian image.

**Fig 2:**
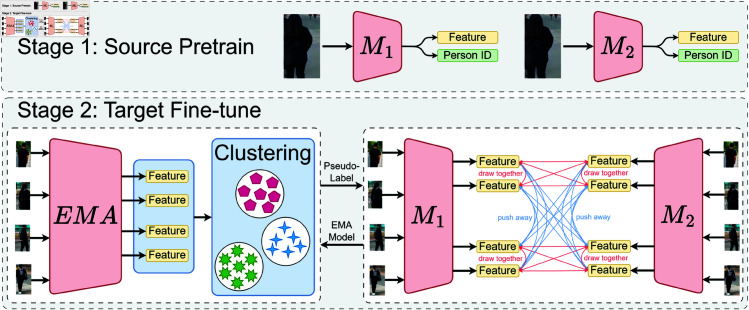
Overall architecture diagram of the model.

The proposed model architecture is illustrated in the Fig 2. The model is designed to learn from the source domain and subsequently transfer knowledge to the target domain, aiming for enhanced person re-identification (ReID) performance. The process is divided into two primary stages: source domain pre-training and target domain fine-tuning. Initially, a pedestrian image from the source domain is input and processed through two feature extraction models, *M*_1_and*M*_2_. Subsequently, fine-tuning is performed on the target domain. We employ Exponential Moving Average (EMA) to smooth the model parameter updates. Furthermore, we cluster the target domain images to generate pseudo-labels. These pseudo-labels facilitate fine-tuning on the target domain, allowing for the adjustment of model parameters to improve accuracy. During fine-tuning, contrastive learning is utilized to aggregate features of the same pedestrian while separating features of different pedestrians.

### Source domain pre-training

Source domain pre-training is used to train the model through the source domain dataset, where images from the source domain dataset are used to learn the feature representation of pedestrians. The input images are processed and features are extracted separately through two sub-networks*M*_1_and*M*_2_, The purpose of each sub-network is to extract features such as body type, clothing, pose etc. from the images for subsequent identity classification.

Suppose the input image is *x*, the representation after feature extraction is $f_{1}=f(x;\theta_{1})$ and $f_{2}=f(x;\theta_{2})$, where $\theta_{1}$ and $\theta_{2}$ are the parameters of the two sub-networks.

Furthermore, we apply the triplet loss function. The core concept of the triplet loss is to select, for each sample, a positive and a negative sample, comparing them to the original sample. The positive sample should be drawn closer to the original sample, while the negative sample should be pushed further away.

The triplet loss function is formulated as in Eq 1.:

$$L_{\text{triplet}}=\sum_{i=1}^{N}\max(0,d(x_{i},x_{\text{pos}})-d(x_{i},x_{\text{neg}})+\alpha )$$ (1)

Where $d(x_{i},x_{\text{pos}})$ denotes the distance between the original and positive samples, $d(x_{i},x_{\text{neg}})$ denotes the distance between the original and negative samples, and α is a constant that denotes the minimum interval between positive and negative samples.

### Fine-tuning of the target domain

The target domain fine-tuning phase focuses on fine-tuning and optimizing the feature representation learned from the source domain using data from the target domain.

#### EMA.

In the target domain fine-tuning process, the EMA technique is used to smooth the parameter updates of the model, and each time the parameters of the model θ are updated, the EMA parameters $\theta_{\text{EMA}}$ are not directly adopted from the current parameters θ but are weighted averages of the historical parameters, so that the model can gradually learn and adapt to the new data distributions in the target domain, reduce the training fluctuations due to the differences between the data in the target domain and the data in the source domain, and obtain a more stable learning process and reduce the influence of target domain label noise. At each training cycle, EMA optimizes the pseudo-labels generated from the clustering step, making the update of pseudo-labels more robust and driving the progress of target domain fine-tuning. The equation is formulated as in Eq 2:

$$\theta_{\text{EMA}}^{t}=\lambda \;\theta_{\text{EMA}}^{t-1}\;+\;(1-\lambda )\;\theta^{t}$$ (2)

where $\theta_{\text{EMA}}^{t}$ is the EMA parameter after the *t*-th iteration, λ is a smoothing factor, usually between 0 and 1, that controls the influence weight of the historical parameters, and $\theta^{t}$ are the model parameters for the current iteration.

#### K-means clustering.

K-means clustering divides the dataset into *K* clusters such that the data points in each cluster are as similar as possible and the clusters are as different from each other as possible. This process groups the samples based on their characteristics and the center of each cluster is called the cluster center *c*_*k*_. The objective of K-means is to minimize the distance of the samples within each cluster, to make the data points within each cluster as close as possible to the center of the cluster. The objective function of K-means is to optimize the partitioning of the clusters by minimizing the distance of the samples to the cluster center, The equation is formulated as in Eq 3:

$$J(\{c_{k}\})=\sum_{k=1}^{K}\sum_{i\in C_{k}}{\left\|f_{i}-c_{k}\right\|}^{2}$$ (3)

where *c*_*k*_ is the centroid of the *k*-th cluster, *C*_*k*_ is the set of samples belonging to the *k*-th cluster, *f*_*i*_ is the eigenvector of the sample *i*, and ${\left\|f_{i}-c_{k}\right\|}^{2}$ is the square of the distance.

During fine-tuning on the target domain, the absence of ground truth labels necessitates the generation of pseudo-labels for target domain samples. We employ K-means clustering for this purpose. Initially, *K* data points are randomly selected as cluster centroids. Each sample is then assigned to the nearest cluster based on its distance to the cluster centroids. The mean of all samples within each cluster is computed, and the cluster centroid is updated to this mean. This process iterates until the cluster centroids stabilize or a maximum number of iterations is reached. Each sample, denoted as *f*_*i*_, is assigned to a cluster *C*_*k*_ and labeled with the corresponding cluster index ${\hat{y}}_{i}=k$. These generated pseudo-labels are subsequently utilized as target domain labels for training models *M*_1_ and *M*_2_.

#### Dynamic confidence threshold adjustment.

To further enhance the reliability of pseudo-labels generated during target domain fine-tuning, we introduce a dynamic confidence threshold adjustment module. This module dynamically adjusts the threshold for selecting pseudo-labeled samples based on the training progress, effectively balancing the trade-off between training stability and adaptability.

In unsupervised person re-identification, clustering algorithms such as K-means are usually used to assign pseudo-labels to unlabeled samples in the target domain. However, due to the domain shift between the source and target domains, the pseudo-labels generated at early training stages are often noisy. Using such noisy labels directly for training can adversely affect the learning process, leading to suboptimal feature representations and poor model generalization.

To address this challenge, this paper incorporates dynamic confidence thresholds into the progressive development of the training process. The potential motivation is to allow the model to initially focus on high-confidence pseudo-labeled samples that are more reliable and less likely to be noisy. As training progresses, the model becomes stronger and the threshold increases gradually, allowing the model to combine more challenging and different samples. This progressive selection strategy not only mitigates the impact of noisy pseudo-labels, but also enhances the model’s adaptability to the target domain.

Let *t* denote the current epoch index (starting from 0) and *T* represent the total number of training epochs. The initial confidence threshold is defined as *p*_0_, with a typical range between 0 and 1. The dynamic confidence threshold at epoch *t*, denoted as *p*(*t*), is formulated as in Eqs 4 and (5):

$$p(t)=\text{clip}(p_{0}+\frac{2}{3}\cdot \frac{au\cdot (6+au)}{6+4\cdot au},\;0,\;1)$$ (4)

$$au=\left(\frac{t}{T}\right)\cdot (e^{\frac{3}{2}(1-p_{0})}-1)$$ (5)

In this formulation, *au* captures the adjustment factor that increases gradually with training epochs. The exponential term $e^{\frac{3}{2}(1-p_{0})}$ ensures that the growth of *au* is sensitive to the choice of the initial threshold *p*_0_. The clip function confines the threshold within the valid range [0, 1].

This design ensures that at the beginning of training (when *t* = 0), the dynamic threshold *p*(*t*) is approximately equal to the initial threshold *p*_0_, allowing the model to select only the most reliable pseudo-labels. As *t* increases, the threshold *p*(*t*) gradually rises, enabling the model to incorporate a wider range of pseudo-labeled samples with varying degrees of confidence. This gradual inclusion of more challenging samples promotes better generalization and robustness.

To validate the effectiveness of different initial threshold values, we conduct extensive experiments with *p*_0_ values set to 0.1, 0.3, 0.5, and 0.7. The experimental results are summarized in Tables 1 and 2. For the DukeMTMC→Market1501 task Table 1, the model achieves the best mAP (80.2) and Rank-1 (91.4) performance at *p*_0_ = 0.1. As *p*_0_ increases, both mAP and Rank-1 decline gradually, indicating that the model struggles with noisy pseudo-labels at higher thresholds. A similar trend is observed in the Market1501→DukeMTMC task Table 2, where *p*_0_ = 0.1 also achieves the highest mAP (68.4) and Rank-1 (81.1). Higher thresholds lead to performance degradation, likely due to insufficient target samples in early training stages that are crucial for model adaptation.

**Table 1:** Analysis of different *p*_0_ values on DukeMTMCTOMarket1501 task.

$p_{0}$	mAP	Rank-1	Rank-5	Rank-10
0.1	80.2	91.4	96.8	97.9
0.3	77.9	90.3	96.1	97.5
0.5	75.6	89.5	95.6	96.9
0.7	72.7	88.2	95.1	96.9

**Table 2:** Analysis of different *p*_0_ values on Market1501TODukeMTMC task.

$p_{0}$	mAP	Rank-1	Rank-5	Rank-10
0.1	68.4	81.1	89.5	92.3
0.3	67.6	79.8	89.2	91.6
0.5	67.8	80.3	89.0	92.3
0.7	65.8	79.4	87.4	89.5

Lower *p*_0_ values allow the model to select a larger portion of the target domain samples at the early stage of training. This provides more diverse data for the model to adapt quickly to the target domain distribution, which is especially important given the significant domain shift between source and target domains. Although including more pseudo-labels at lower thresholds may introduce some noisy samples, the self-training mechanism with dynamic threshold adjustment gradually corrects mislabeled samples during the training process. This mechanism effectively mitigates the impact of label noise while retaining the benefits of diverse training data.In contrast, higher *p*_0_ values, while more conservative, tend to overly restrict the training samples at the beginning. As a result, the model only learns from a small subset of highly confident samples, leading to overfitting and limiting its ability to generalize to the broader target domain distribution. Therefore, despite the potential risk of noisy labels, lower *p*_0_ values provide a better trade-off between learning stability and sample diversity, enabling the model to achieve superior performance in challenging domain adaptation scenarios.

#### Contrastive learning.

In the target domain fine-tuning phase, in addition to supervised training using pseudo-labels, the model further optimizes the feature representation through contrast learning. Specifically, the features extracted by the *M*_1_ and *M*_2_ networks are optimized by contrast loss. Contrast learning here helps the model to make the same features in the target domain more discriminative by minimizing the feature distance between similar samples and maximizing the feature distance between dissimilar samples. The contrast learning loss function is as in Eq (6):

$$L_{\text{contrast}}=\frac{1}{4}[L_{t1↔\text{ema}}+L_{t2↔\text{ema}}+L_{t1↔t1}+L_{t2↔t2}]$$ (6)

$$L_{t1↔\text{ema}}=-\frac{\sum_{i}𝕀(n_{i}^{t1↔\text{ema}}>0)\log(\frac{\sum_{j}\exp(s_{ij}^{t1,\text{ema}})\cdot M_{ij}^{t1,\text{ema}}}{\sum_{k}\exp(s_{ik}^{t1,\text{ema}})+\epsilon }+\epsilon )}{\sum_{i}𝕀(n_{i}^{t1↔\text{ema}}>0)+\epsilon }$$ (7)

$$L_{t2↔\text{ema}}=-\frac{\sum_{i}𝕀(n_{i}^{t2↔\text{ema}}>0)\log(\frac{\sum_{j}\exp(s_{ij}^{t2,\text{ema}})\cdot M_{ij}^{t2,\text{ema}}}{\sum_{k}\exp(s_{ik}^{t2,\text{ema}})+\epsilon }+\epsilon )}{\sum_{i}𝕀(n_{i}^{t2↔\text{ema}}>0)+\epsilon }$$ (8)

$$L_{t1↔t1}=-\frac{\sum_{i}𝕀(n_{i}^{t1↔t1}>0)\log(\frac{\sum_{j\neq i}\exp(s_{ij}^{t1,t1})\cdot M_{ij}^{t1,t1}}{\sum_{k}\exp(s_{ik}^{t1,t1})+\epsilon }+\epsilon )}{\sum_{i}𝕀(n_{i}^{t1↔t1}>0)+\epsilon }$$ (9)

$$L_{t2↔t2}=-\frac{\sum_{i}𝕀(n_{i}^{t2↔t2}>0)\log(\frac{\sum_{j\neq i}\exp(s_{ij}^{t2,t2})\cdot M_{ij}^{t2,t2}}{\sum_{k}\exp(s_{ik}^{t2,t2})+\epsilon }+\epsilon )}{\sum_{i}𝕀(n_{i}^{t2↔t2}>0)+\epsilon }$$ (10)

$L_{\text{contrast}}$ denotes the loss function for comparison learning. The core concept revolves around the design of a contrastive learning-based loss function, applied across two dimensions: inter-model (contrastive learning between different models) and intra-model (contrastive learning within the same model). This design ensures consistency in learning between the model and its historical weights, thereby enhancing the discriminative power of the embedded representations within a single model. Furthermore, the inter-model contrastive approach contributes to improved robustness and generalization capabilities.

In in Eq (7), where, *i*: Sample index (anchor sample). *j*: Index of the other samples currently used for comparison (positive and negative samples). *k*: Index of all candidate samples used in the denominator normalization term. 𝕀(·): Indicator function, returns 1 if the condition inside is true, 0 otherwise. $n_{i}^{t1↔ema}$: The number of positive samples for anchor *i* between model *t*1 and its EMA branch. $s_{ij}^{t1,ema}$: The similarity score between anchor *i* in model *t*1 and sample *j* in EMA branch. $M_{ij}^{t1,ema}$: Label indicator, 1 if *i* and *j* belong to the same identity (positive pair), 0 otherwise. ϵ: A small constant for numerical stability.

Other terms ($L_{t2↔ema}$, $L_{t1↔t1}$, and $L_{t2↔t2}$ Eqs (8)–(10)) follow the same formulation but differ in model branches and index definitions.

The four sub-loss components correspond to:


$$L_{t1↔ema}:\text{ Comparison of model outputs with their EMA branches.}$$

$$L_{t2↔ema}:\text{ Comparison of model outputs with their EMA branches.}$$

$$L_{t1↔t1}:\text{ Comparison between samples within model }t1\text{ itself.}$$

$$L_{t2↔t2}:\text{ Comparison between samples within model }t2\text{ itself.}$$

**Fig 3:**
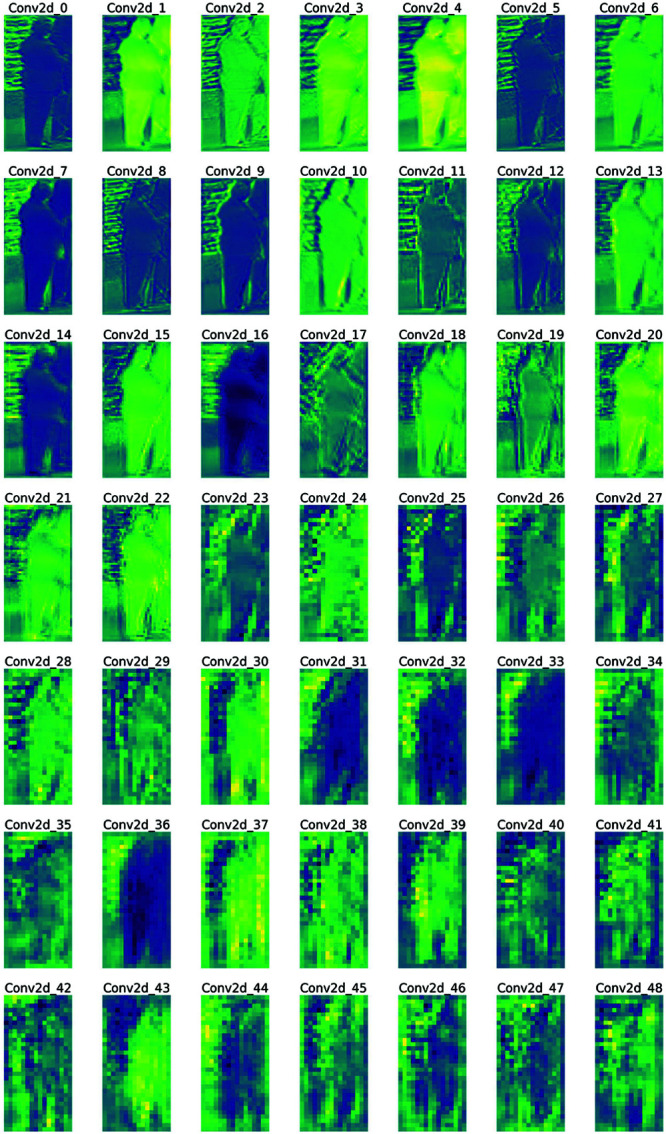
resnet50featuremaps.

This contrastive learning loss function design simultaneously balances representation consistency and discriminability. By introducing EMA-guided cross-model supervision, the model can achieve a more stable and smooth representation learning. The self-contrastive learning within the model enhances its ability to distinguish between different identities. The four sub-loss terms together construct a stable, symmetric, and structured representation learning framework, significantly improving performance and robustness in the cross-domain person re-identification task. In the overall target domain fine-tuning stage, due to the difference in feature distribution between the source and target domains, relying solely on the traditional classification loss may not be able to effectively adapt to the data distribution in the target domain. Through contrast learning, the model cannot only learn effective feature representations on the source domain but also optimize the target domain data to enhance the recognition ability of the target domain. The pseudo-labels generated by K-means clustering provide a reference label for each sample, and contrast learning further optimizes the effectiveness of these pseudo-labels. By utilizing contrastive learning, the model can effectively bring feature representations of similar samples closer together and push feature representations of different samples apart in the target domain, thereby improving person re-identification accuracy in the target domain. The following Fig 3 illustrates feature visualization.

### Total modeled losses

$$L_{\text{total}}=\;\left(L_{\text{CE},1}+L_{\text{CE},2}\right)(1-w_{\text{CE},\text{soft}})\;+\left(L_{\text{tri},1}+L_{\text{tri},2}\right)(1-w_{\text{tri},\text{soft}})\;+L_{\text{CE},\text{soft}}\cdot w_{\text{CE},\text{soft}}+L_{\text{tri},\text{soft}}\cdot w_{\text{tri},\text{soft}}\;+L_{\text{contrast}}\cdot w_{\text{contrast}}$$ (11)

Cross-Entropy Loss: $L_{\text{CE},1}$ and $L_{\text{CE},2}$ are the cross-entropy losses computed by the CrossEntropyLabelSmooth function comparing the two models concerning the target label.

Triplet Loss: $L_{\text{tri},1}$ and $L_{\text{tri},2}$ are triplet losses computed via the TripletLoss function, based on the outputs of the two models. Soft Cross-Entropy Loss: $L_{\text{CE,soft}}$ is the soft cross-entropy loss calculated using the KL dispersion, comparing the difference between the model output and the output of the EMA (Exponentially Weighted Moving Average) model.

Soft Triplet Loss: $L_{\text{tri,soft}}$ is the soft triplet loss calculated using the EMA model, taking into account the difference between the model output and the EMA model output.

Contrastive Loss: $L_{\text{contrast}}$ is the contrastive loss computed similarity between positive sample pairs and optimizes the model by contrasting the similarity of negative sample pairs. Specifically, a positive sample pair consists of two images of the same person, and the model is trained to minimize the distance between these positive pairs while maximizing the distance between negative pairs.

Weighting parameters:

$w_{\text{CE,soft}}$: Weights for soft cross-entropy losses

$w_{\text{tri,soft}}$: Weighting of soft triplet losses

$w_{\text{contrast}}$: Weighting of contrastive losses

Combinations of these loss functions Eq (11) are used to train the model to improve the model’s ability to learn different features and sample relationships.

## Experimental results and analyses

### Datasets and assessment

The Market-1501 dataset was released by the Computer Vision Lab at Shanghai Jiao Tong University, specifically designed for Personre-identification and domain adaptation research. This dataset contains images of pedestrians captured by six different cameras, simulating real-world scenarios where pedestrians are recognized across multiple camera views. The dataset includes data for 1,501 unique pedestrians, with each pedestrian having at least six images captured from different angles, totaling 32,668 images. The training set consists of 751 pedestrians and approximately 12,936 images, while the test set contains 750 pedestrians and around 19,732 images. Additionally, there are 3,368 query images used to evaluate the model’s performance. The DukeMTMC dataset, provided by the Computer Vision Lab at Duke University, includes images from eight different cameras. This dataset contains a total of 36,411 images of 1,404 unique pedestrians, with each pedestrian having at least two images captured from different cameras. The training set consists of 702 pedestrians and 16,522 images, while the test set contains 702 pedestrians and 19,763 images. The query set includes 2,228 images. The situation of the dataset is shown in Table 3.

**Table 3:** Dataset characteristics.

Characterization	Market1501	DukeMTMC
**Number of pedestrians**	1501 pedestrians	1501 pedestrians
**Number of images**	32668 images	36411 images
**Number of cameras**	6 cameras	8 cameras
**Training set pedestrians**	751 pedestrians	702 pedestrians
**Test set pedestrians**	750 pedestrians	702 pedestrians
**Query set images**	3368 images	2228 images

Table notes: Summary of dataset statistics for Market1501 and DukeMTMC, including pedestrian counts, image counts, and camera details.

The metrics for evaluating the performance of the model we use mAP and Rank-1, Rank-5, and Rank-10. mAP is the average of Average Precision (AP). AP measures the proportion of correctly matched samples in the retrieval results. mAP averages the APs of all the queries, which reflects the retrieval ability of the model as a whole. Rank-1, Rank-5, and Rank-10 indicate the presence of correct matches in the top 1, top 5, and top 10 positions of the retrieval results, respectively. These metrics reflect the model’s ability to recognize different retrieval ranges.

### Experimental details

For the network architecture we used ResNet50, which was pre-trained on ImageNet, and in order to test them on adapted scenarios, we chose one of the datasets as the source domain and the other as the target domain. Pytorch was used as the framework, Pytorch version 1.7.0 was used, a TITANXpGPU was used for training and a TITANXpGPU was used for testing. Parameter-wise after debugging we chose the contrast loss weight of 0.01 as the best value.

### Ablation experiments

#### Comparison of different loss functions.

To achieve robust representational learning of the model under diverse structures or multiple processing conditions, we construct a series of contrast loss functions to constrain the representational consistency of the model output under different semantic scenarios or different structural perturbations. Unlike traditional contrast learning tasks that focus on discriminative properties between image pairs, this series of designs focuses more on model output stability, consistency across structures, and coordination of internal representation distributions.

From the perspectives of “modeling the consistency between models” and “modeling the stability of the internal structure of the model”, four contrast loss functions are designed in turn, and they are applied to the core task model one by one for ablation experiments to explore the impact of different contrast paradigms on the overall performance of the model. Paradigms on the overall performance of the model.

$$L_{1}=\frac{1}{2}[-\frac{\sum_{i}𝕀(n_{i}^{1↔1}>0)\log(\frac{\sum_{j\neq i}\exp(s_{ij}^{11})\cdot M_{ij}^{11}}{\sum_{k}\exp(s_{ik}^{11})+\epsilon }+\epsilon )}{\sum_{i}𝕀(n_{i}^{1↔1}>0)+\epsilon }\;\;-\frac{\sum_{i}𝕀(n_{i}^{2↔2}>0)\log(\frac{\sum_{j\neq i}\exp(s_{ij}^{22})\cdot M_{ij}^{22}}{\sum_{k}\exp(s_{ik}^{22})+\epsilon }+\epsilon )}{\sum_{i}𝕀(n_{i}^{2↔2}>0)+\epsilon }]$$ (12)

To improve the robustness of the models under structural perturbations, we design this function to enhance the semantic consistency learning within the models by comparing the internal attention of model 1 and model 2 with “exclude self”. The original intention of this design is that each model should maintain a stable semantic focus within its structure if the pseudo-labels are credible. However, experiments show that this design fails to significantly improve performance due to the conflicting goals of internal contrast and cross-model contrast. The internal comparison attempts to optimize the fine-grained distinction of features within each model, whereas the cross-model comparison places more emphasis on the consistency of features across models. Since the loss function lacks an effective balance between the two objectives, it is easy to cause the models to focus too much on their features and ignore cross-model semantic alignment during training, which reduces the overall representation quality and generalization ability.

$$L_{2}=\frac{1}{3}[-\frac{\sum_{i}𝕀(n_{i}^{1↔2}>0)\log(\frac{\sum_{j\neq i}\exp(s_{ij}^{12})\cdot M_{ij}^{12}}{\sum_{k}\exp(s_{ik}^{11})+\epsilon }+\epsilon )}{\sum_{i}𝕀(n_{i}^{1↔2}>0)+\epsilon }\;\;-\frac{\sum_{i}𝕀(n_{i}^{1↔1}>0)\log(\frac{\sum_{j\neq i}\exp(s_{ij}^{11})\cdot M_{ij}^{11}}{\sum_{k}\exp(s_{ik}^{11})+\epsilon }+\epsilon )}{\sum_{i}𝕀(n_{i}^{1↔1}>0)+\epsilon }\;\;-\frac{\sum_{i}𝕀(n_{i}^{2↔2}>0)\log(\frac{\sum_{j\neq i}\exp(s_{ij}^{22})\cdot M_{ij}^{22}}{\sum_{k}\exp(s_{ik}^{22})+\epsilon }+\epsilon )}{\sum_{i}𝕀(n_{i}^{2↔2}>0)+\epsilon }]$$ (13)

By improving it we design a loss function that combines cross-model contrast with intra-model contrast. The design consists of three items: firstly, cross-model comparison (Model 1 vs. Model 2) to strengthen the model’s consistent understanding of pseudo-labels, which in turn enhances the semantic alignment capability; and secondly, the introduction of self-internal comparisons between Model 1 and Model 2 to enhance the feature differentiation and focusing capability within the respective models. The design was initially intended to incorporate multi-view feature learning to simulate a more realistic prediction of structural diversity. However, the experimental results show that the method still falls short in terms of practical effectiveness. While internal comparisons help to refine semantic structures, there is an optimization conflict with the goal of cross-model comparisons. Models may over-optimize their structural representations during training, thus weakening the learning of cross-model semantic consistency and leading to a decrease in the overall representation ability. In addition, the fixed weight design of contrast items fails to dynamically balance these two types of objectives, further limiting model performance.

$$L_{3}=-\frac{\sum_{i}𝕀(n_{i}>0)\log(\frac{\sum_{j}\exp(s_{ij})\cdot M_{ij}}{\sum_{k}\exp(s_{ik})+\epsilon }+\epsilon )}{\sum_{i}𝕀(n_{i}>0)+\epsilon }$$ (14)

To simplify the training structure and reduce redundant computation, we design a lightweight contrast loss function, which omits cross-model alignment and intra-model self-comparisons, and focuses on the semantic similarity between samples. However, experiments show that this simplified design improves training stability under certain conditions, but its overall performance still has obvious limitations. First, the loss function completely abandons modeling the structural stability of the model and lacks smoothing references such as EMA, resulting in feature representations that are prone to drift in the face of long-period training or structural perturbations. Second, it performs poorly in multi-model collaborative learning scenarios due to the lack of semantic alignment between cross-model outputs, which makes it difficult to capture the consensus expression between models for the same pseudo-label. In addition, the absence of an internal contrast term also means that the robustness of the model to its attentional structure cannot be constrained, and the training process is prone to attentional focus drift. Overall, despite the simplicity of this loss function training process, its ability to cope with complex structural perturbations and pseudo-labeling interference is insufficient, and it is not suitable for scenarios that require high generalization ability.


$$L_{\text{contrast}}=\frac{1}{4}\left[L_{t1↔ema}+L_{t2↔ema}+L_{t1↔t1}+L_{t2↔t2}\right]$$

The detailed definitions of each sub-loss term ($L_{t1↔ema}$, $L_{t2↔ema}$, $L_{t1↔t1}$, and $L_{t2↔t2}$ as Eqs (7)–(10)) are provided in the Contrastive Learning. Based on the above considerations, we designed the final contrastive loss function as in Eq (6). This loss function integrates both cross-model contrast and intra-model consistency modeling mechanisms. It also introduces an EMA (Exponential Moving Average) model as a semantic reference for stable structures, forming a comprehensive structure of four sub-losses. Compared to the previous two designs, this design provides rich contrast signals while introducing smooth feature trajectories, effectively mitigating drastic fluctuations in model output. In contrast to the third design, this method introduces a structurally stable reference, enabling feature learning with stronger global consistency. Specifically, through the contrast between the model and the EMA, we achieve a unified constraint of long-term stability and short-term dynamics, significantly enhancing the continuity, consistency, and discriminative power of feature representation during the training process. Table 4 shows a comparison of the performance of each function.

**Table 4:** mAP Values of different functions.

Loss	DukeMTMC-Market1501	Market1501-DukeMTMC
*L*_1_	74.9	67.5
*L*_2_	75.1	67.6
*L*_3_	75.4	67.5
*L*_*contrast*_	**79.0**	**67.9**

Table notes: Comparison of mAP values for different loss functions on DukeMTMC-Market1501 and Market1501-DukeMTMC datasets.

#### Comparison of metrics.

(1) Loss ce

**Fig 4:**
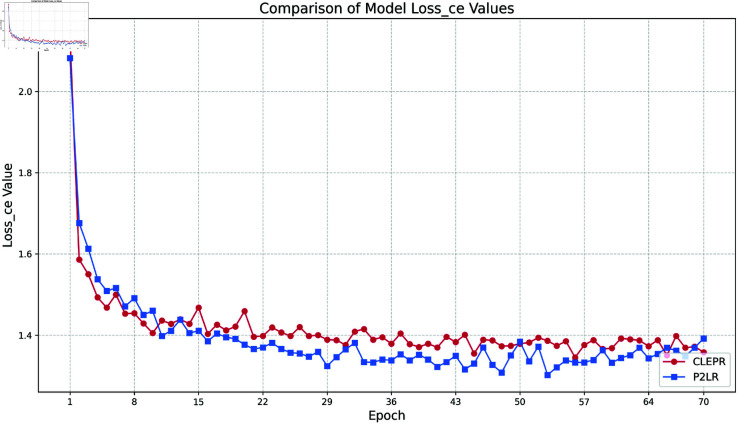
DukeMTMCTOMarket1501 loss ce.

In Fig 4, the CLEPR method decreases its loss value more rapidly than P2LR in the early stage of training, implying that CLEPR is able to fit the data more efficiently in the initial training stage, and in the later stage of training, the loss curve of CLEPR is relatively smooth and stable, and the final loss value of CLEPR is lower than that of P2LR, which has better optimization effect.

**Fig 5:**
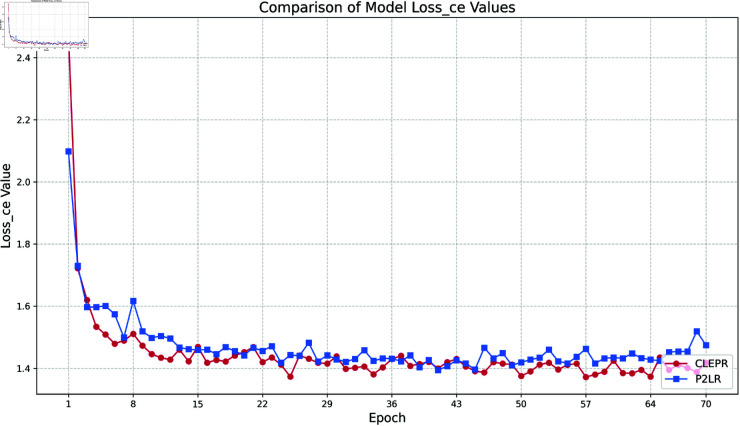
Market1501TODukeMTMC loss ce.

In Fig 5, the loss value of the CLEPR method decreases faster in the early stage of training, showing more rapid convergence, and thereafter the loss value tends to level off gradually with less fluctuation, and the loss value in the late stage of training is much lower than that of P2LR, which makes the optimization effect better.

(2) Loss ce soft

**Fig 6:**
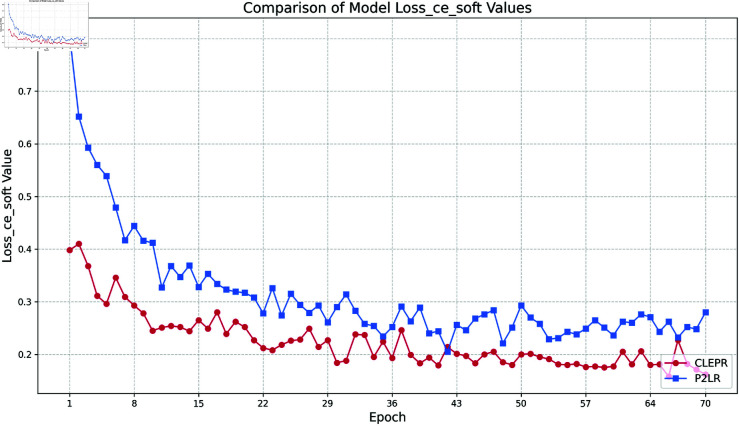
DukeMTMCTOMarket1501 loss ce soft.

In Fig 6, the CLEPR method always maintains a low loss value during the training process, and with the training, the loss value decreases steadily and eventually levels off, and the loss value fluctuation of the whole process is more stable compared to that of P2LR, which has a stronger generalization ability.

**Fig 7:**
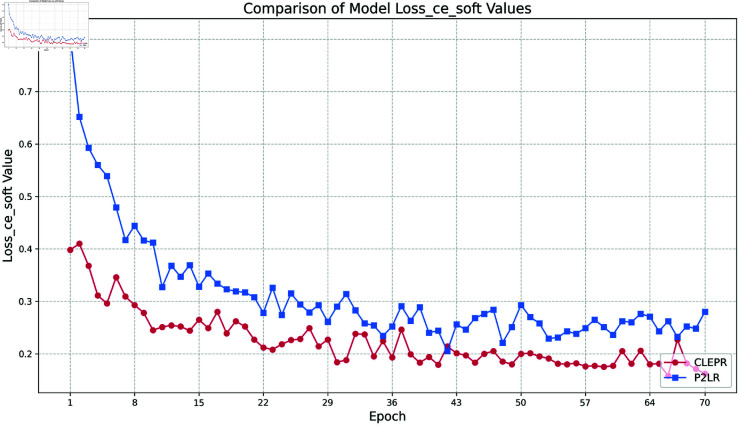
Market1501TODukeMTMC loss ce soft.

In Fig 7, the CLEPR method decreases rapidly from the 1st to the 8th epoch at the beginning of training, and the loss value gradually tends to stabilize as the training progresses, showing good stability and continuous optimization effect. P2LR, on the other hand, has large fluctuations in the training process, and the loss value fails to continue to decline steadily. The overall process loss value of CLEPR is lower than that of P2LR.

(3) Loss contrast

**Fig 8:**
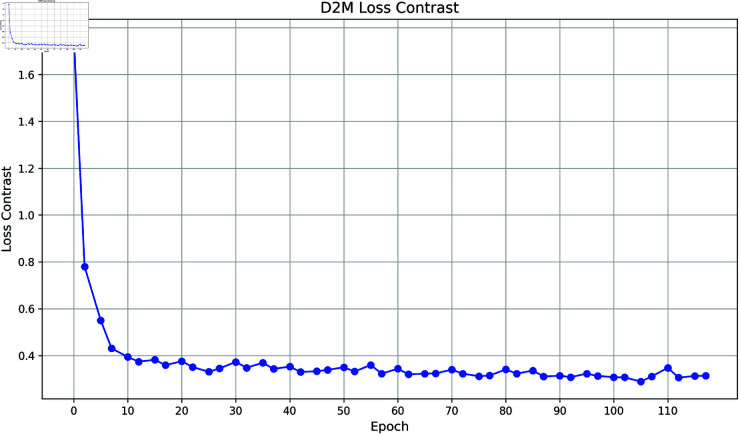
DukeMTMCTOMarket1501 loss contrast.

**Fig 9:**
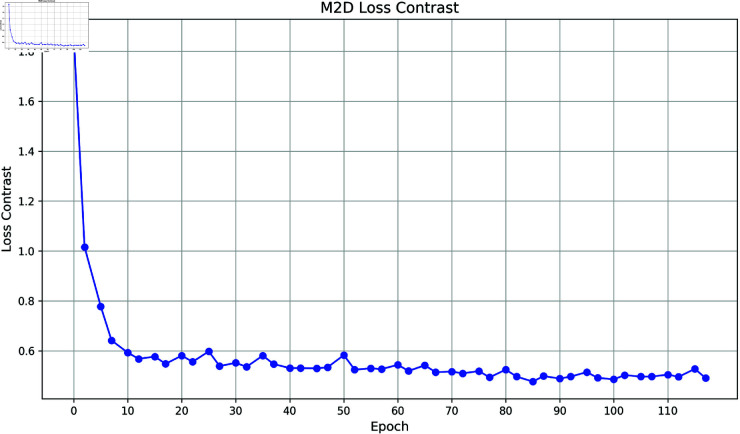
Market1501TODukeMTMC loss contrast.

In Figs 8 and 9, loss contrast is the unique contrast loss function after adding contrast learning to CLEPR, the contrast loss function decreases rapidly in the early stage of training, indicating that CLEPR the model can adapt to the data quickly and reduce the error significantly during the learning process, and the loss value tends to stabilize in the late stage of training, indicating that stable optimization has been achieved. After adding contrast learning in the CLEPR model, the optimization of the loss function includes not only the traditional classification loss but also the contrast loss, thus enhancing the model’s ability to distinguish between features and the stability of learning.

(4) mAP

**Fig 10:**
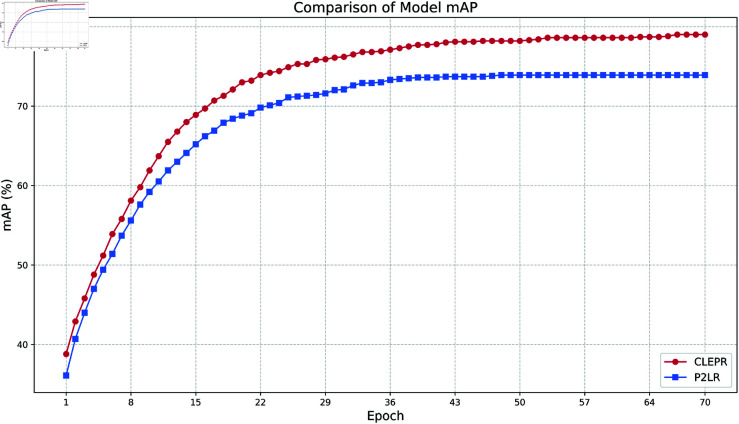
DukeMTMCTOMarket1501 mAP.

In Fig 10, CLEPR can rapidly improve its accuracy in the early stages of training, especially within the first 10 Epochs, showing very fast convergence. Being able to start getting better accuracy in a shorter period shows that the model can learn the features in the data effectively and adapt to the training set quickly. In the later stages of training, CLEPR continues to steadily increase its accuracy with a very smooth curve, which indicates that the model can maintain a better optimization over a long period of training, showing strong stability, while P2LR saturates in the later stages.

**Fig 11:**
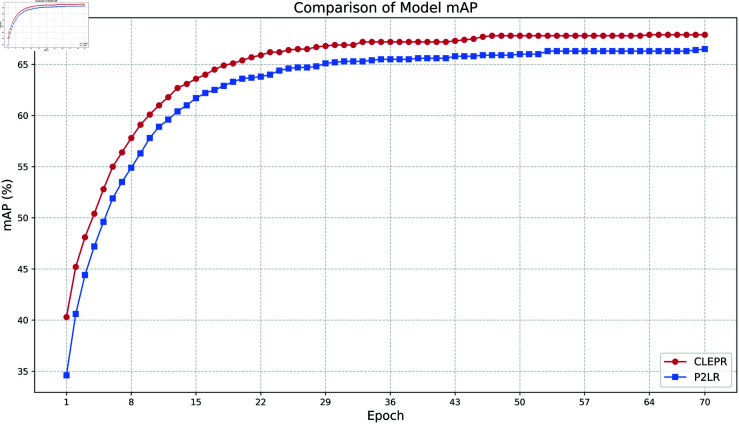
Market1501TODukeMTMC mAP.

In Fig 11, CLEPR also shows a fast convergence speed, especially within the first 20 Epochs of training, the accuracy increases rapidly. The early training of CLEPR can effectively learn the data features and improve its performance in a shorter period, which proves that the model is more capable of learning the data in the cross-domain task. In the later stages of training, the value of CLEPR maintains a smooth and high growth trend, which is relatively smooth without too much fluctuation showing better training stability, avoiding overfitting or instability.

### Feature space visualization

To further validate the effectiveness of the proposed method in learning discriminative feature representations in the target domain, this study provides comprehensive t-SNE visualizations of the target domain features under different clustering configurations. Such visualizations are crucial for intuitively understanding the model’s ability to enhance intra-class compactness and inter-class separability in the feature space—both of which are essential for achieving robust cross-domain person re-identification performance.

**Fig 12:**
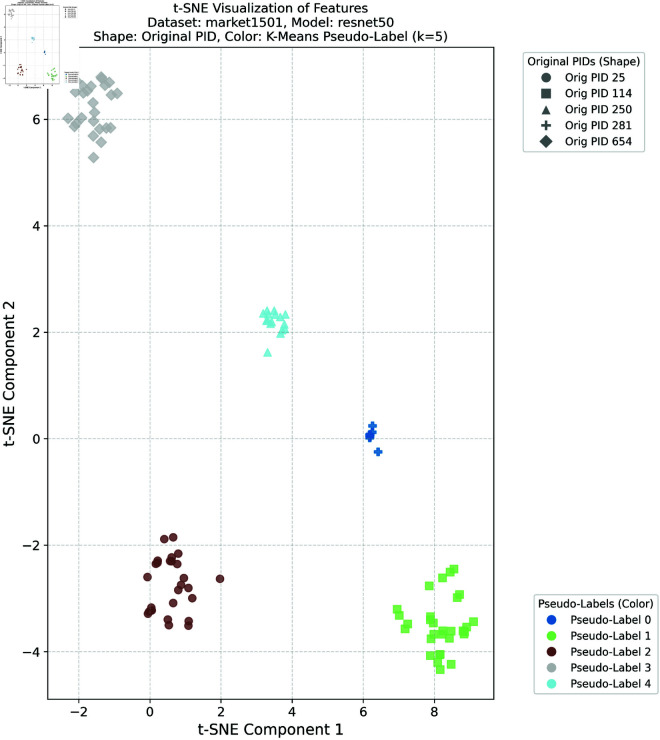
t-SNE Market1501 k = 5.

**Fig 13:**
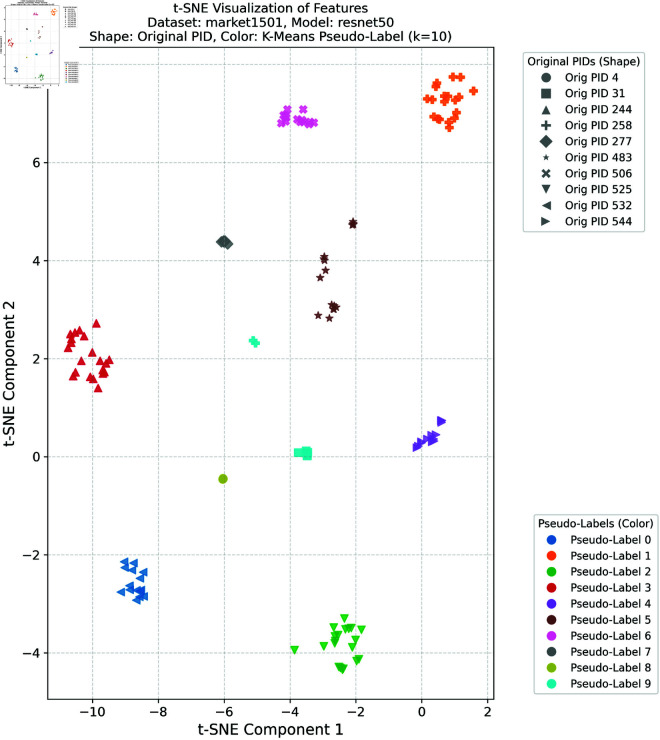
t-SNE Market1501 k = 10.

**Fig 14:**
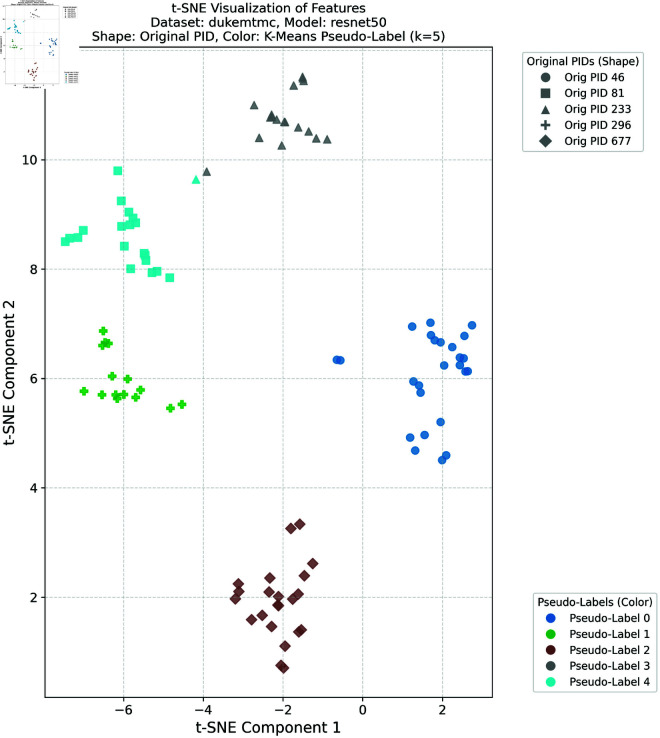
t-SNE DukeMTMC k = 5.

**Fig 15:**
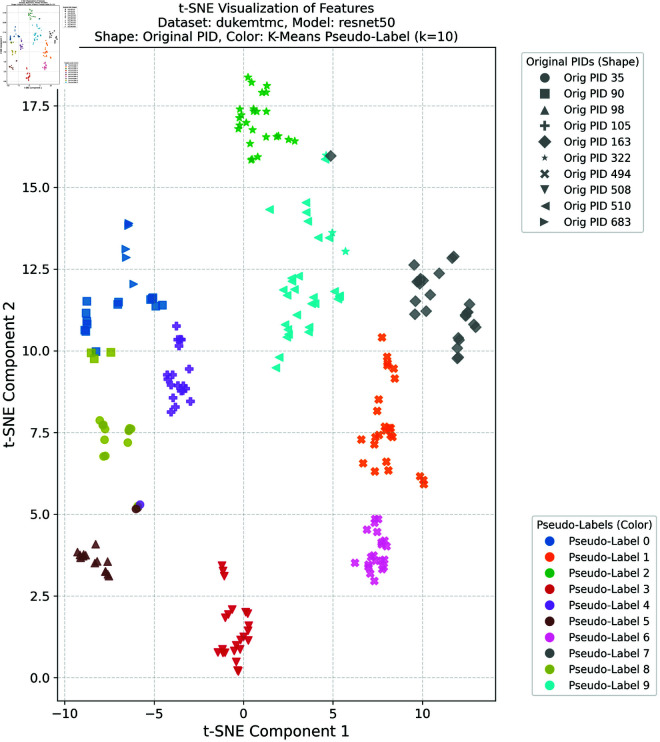
t-SNE DukeMTMC k = 10.

In this experiment, we selected the DukeMTMC dataset as the source domain and the Market-1501 dataset as the target domain, as well as the Market-1501 dataset as the source domain and the DukeMTMC dataset as the target domain, respectively. K-means clustering was applied to the target domain features with four different cluster numbers: *k* = 5, *k* = 10, *k* = 20, and *k* = 30. These cluster numbers were chosen to evaluate the robustness and generalization ability of the proposed framework under different clustering granularities and pseudo-label noise levels. For the configuration with DukeMTMC as the source domain and Market-1501 as the target domain, the t-SNE visualizations corresponding to *k* = 5 and *k* = 10 are presented in Fig 12 and Fig 13, respectively, while the results for *k* = 20 and *k* = 30 are provided in S1 Fig and S2 Fig. For the configuration with Market-1501 as the source domain and DukeMTMC as the target domain, the t-SNE visualizations for *k* = 5 and *k* = 10 are shown in Fig 14 and Fig 15, respectively, with the results for *k* = 20 and *k* = 30 available in S3 Fig and S4 Fig. In these figures, the shapes represent the original pedestrian identities (PIDs) of the DukeMTMC dataset, while the colors represent the pseudo-labels assigned by K-means clustering. This dual-annotation visualization allows us to analyze both the consistency between the extracted features and the ground-truth PIDs, and to evaluate the effectiveness of pseudo-label assignments in aligning with the true identity distribution.

Overall, these t-SNE visualizations complement the quantitative results presented earlier by providing intuitive and compelling evidence that the proposed framework effectively constructs discriminative feature spaces in the target domain. The alignment between visual analysis and quantitative performance further confirms the superior performance of the proposed method in cross-domain person re-identification, indicating its ability to effectively bridge the domain gap and mitigate pseudo-label noise under various clustering conditions.

### Performance comparison

To further demonstrate the effectiveness of this paper’s method, Table 3 presents the performance metrics of our method compared with several state-of-the-art methods for cross-domain person re-identification in recent years. These comparison methods include generative adversarial network-based approaches (e.g., HHL [23]), cluster-based pseudo-labeling frameworks (e.g., BUC [25], UDAP [27], SILC [29]), and self-training with progressive representation enhancement (PREST [30]). The table also includes mutual training and consistency-enhancing methods (e.g., NRMT [32], DCML [33]) that leverage dual models and pseudo-label refinement to improve robustness to noisy labels. Additionally, methods focusing on local feature learning and body part modeling (e.g., SSG [28]) and other advanced techniques (e.g., MEB-Net [36], CNRN [37]) are included for comparison. The performance is evaluated using Market1501 and DukeMTMC as the source domains and DukeMTMC and Market1501 as the target domains, respectively, and the results are shown in Table 5.

**Table 5:** Performance comparison of different methods.

Method		DukeMTMC-Market1501				Market1501-DukeMTMC			
Name	Reference	mAP	Rank1	Rank5	Rank10	mAP	Rank1	Rank5	Rank10
HHL [23]	ECCV18	31.4%	62.2	78.8	84.0	27.2%	46.0	61.0	66.7
CSCL [24]	ECCV18	35.6%	64.7	80.2	85.6	30.5%	51.5	66.7	71.7
BUC [25]	AAAI19	38.3%	66.2	79.6	84.5	27.5%	47.4	62.6	68.4
ECN [26]	CVPR19	43.0%	75.1	87.6	91.6	40.4%	63.3	75.8	80.4
UDAP [27]	NeurIPS20	53.7%	75.8	80.5	93.2	49.0%	68.4	80.1	83.5
SSG [28]	ICCV19	58.3%	80.0	90.0	92.4	53.4%	73.0	80.6	83.2
SILC [29]	KBS21	61.8%	80.7	90.1	93.0	50.3%	68.5	80.2	85.4
PREST [30]	IEEE21	62.4%	82.5	92.1	94.9	56.1%	74.4	83.7	85.9
AD-Cluster [31]	CVPR20	68.3%	86.7	94.4	96.5	54.1%	72.6	82.5	85.5
NRMT [32]	ECCV20	71.7%	87.8	94.6	96.5	62.2%	77.8	86.9	89.5
DCML [33]	ECCV20	72.3%	88.2	94.9	96.4	63.5%	79.3	86.7	89.5
MMT-500 [34]	ICCR20	71.2%	87.7	94.9	96.9	63.1%	76.8	88.0	92.2
P2LR [35]	AAAI22	73.9%	87.3	94.5	96.6	66.5%	78.6	88.6	91.1
MEB-Net [36]	ECCV20	76.0%	89.9	96.0	97.5	66.1%	79.6	88.3	92.2
CNRN [37]	AAAI21	78.1%	91.9	96.1	97.8	69.1%	82.0	90.7	93.5
**OURS**	**-**	**79.0%**	**91.4**	**96.2**	**97.5**	**67.9%**	**81.4**	**89.7**	**92.3**

Table notes: Comparison of different cross-domain person re-identification methods on DukeMTMC-Market1501 and Market1501-DukeMTMC datasets.

As shown in the Table 5, this paper’s method achieves 91.4% and 81.4% Rank-1 accuracy, as well as 79.0% and 67.9% mAP for DukeMTMC migration to Market1501 and Market1501 migration to DukeMTMC, respectively. Compared with P2LR, for DukeMTMC migration to Market1501, the Rank-1, and mAP of this paper’s method are improved by 4.1 percentage points and 5.1 percentage points, respectively; for Market1501 migration to DukeMTMC, the Rank-1 and mAP of this paper’s method are improved by 2.8 percentage points and 1.4 percentage points, respectively.

### Additional experiments

To further verify the effectiveness and generalization capabilities of this method, in addition to performance evaluation on mainstream datasets such as Market1501 and DukeMTMC, cross-domain experiments were conducted with Market1501 and DukeMTMC on additional CUHK03 and PersonX datasets. These datasets include different shooting environments, perspectives and lighting conditions, allowing a more comprehensive examination of the model’s adaptability and robustness in complex scenarios. Through testing of these data sets, the results show that the method proposed in this paper not only achieves excellent performance on the main data sets, but also shows excellent performance on the new data sets, significantly better than the existing comparison methods. This fully proves the robustness of the model and its strong cross-scene migration capabilities, and further verifies the popularization and practical value of this method in actual scenarios. The experimental results are shown in Table 6, and the corresponding mAP comparison results can be found in Supporting Information S5–S10 Fig.

**Table 6:** Performance comparison of different methods on cross-domain person re-identification tasks (mAP, Rank-1, Rank-5, Rank-10).

Task Direction	Method	mAP	Rank-1	Rank-5	Rank-10
CUHK03→DukeMTMC	P2LR	64.2	78.1	87.5	90.4
CUHK03→DukeMTMC	CLEPR	66.1	80.1	88.4	91.2
CUHK03→Market1501	P2LR	79.1	91.0	96.6	97.8
CUHK03→Market1501	CLEPR	81.0	91.5	96.8	98.0
PersonX→DukeMTMC	P2LR	64.8	77.9	87.9	90.6
PersonX→DukeMTMC	CLEPR	66.9	79.5	88.8	92.3
PersonX→Market1501	P2LR	62.1	79.7	88.7	91.8
PersonX→Market1501	CLEPR	66.9	79.5	88.8	92.3
DukeMTMC→CUHK03	P2LR	16.0	13.3	26.2	33.2
DukeMTMC→CUHK03	CLEPR	19.8	18.1	31.8	39.1
Market1501→CUHK03	P2LR	19.6	17.4	30.5	40.7
Market1501→CUHK03	CLEPR	31.3	29.4	47.0	56.9

Table Notes: This table compares the mAP, Rank-1, Rank-5, and Rank-10 performance of two methods on DukeMTMC-Market1501 and Market1501-DukeMTMC datasets.

## Conclusion

In unsupervised cross-domain Person re-identification, the quality of pseudo-labels is crucial, and the accuracy of pseudo-labels directly determines the learning effect of the model. In this paper, we introduce a contrast learning approach to enhance the quality of pseudo-labels. Contrast learning helps the model to better capture the intrinsic similarity between similar samples, e.g., different images of the same person should be closer in the feature space, while images of different people should be farther away. This approach makes the pseudo-labels more accurate in generating, which in turn provides the model with higher-quality supervised signals. Contrast learning also reinforces the differentiation of features by constructing pairs of positive and negative samples. For each target sample, we not only consider it by itself but also enhance the learning by comparing it with other samples. In addition, the pseudo-labeling approach enhanced by contrastive learning can bridge the gap between the target domain and the source domain, which can effectively alleviate the problem of labeling difficulty in unsupervised domain adaptation, especially when data labeling is scarce. However, this study still has shortcomings, for example, especially in complex scenarios, changes in the appearance of pedestrians may lead to a high false-positive rate in comparison learning, thus affecting the accuracy of pseudo-labeling. To improve this, future research could consider introducing more refined contrast learning methods, such as self-supervised learning and intra-class clustering, to further enhance the model’s ability to adapt to complex scenes and improve the generation of pseudo-labels.

## Supporting information

S1 Fig t-SNE Market1501 k = 20. (TIF)

S2 Fig t-SNE Market1501 k = 30. (TIF)

S3 Fig t-SNE DukeMTMC k = 20. (TIF)

S4 Fig t-SNE DukeMTMC k = 30. (TIF)

S5 Fig CUHK03TODukeMTMC mAP. (TIF)

S6 Fig CUHK03TOMarket1501 mAP. (TIF)

S7 Fig PersonXTODukeMTMC mAP. (TIF)

S8 Fig PersonXTOMarket1501 mAP. (TIF)

S9 Fig DukeMTMCTOCUHK03 mAP. (TIF)

S10 Fig Market1501TOCUHK03 mAP. (TIF)
