# Correction: Contrastive learning enhanced pseudo-labeling for unsupervised domain adaptation in person re-identification

**DOI:** 10.1371/journal.pone.0337650

**Published:** 2025-11-25

**Authors:** Xuemei Bai, Yuqing Zhang, Chenjie Zhang, Zhijun Wang

The Funding statement for this article is incorrect. The correct Funding statement is as follows: This research was supported by the Department of Science and Technology of Jilin Province, China, project number: 20240302089GX. The authors declare no conflict of interest.
