# Supplementary figures and images for: Contrastive learning enhanced pseudo-labeling for unsupervised domain adaptation in person re-identification
Source: PLoS One. 2025 Jul 14;20(7):e0328131. doi: 10.1371/journal.pone.0328131 (PMC12258581; doi:10.1371/journal.pone.0328131)

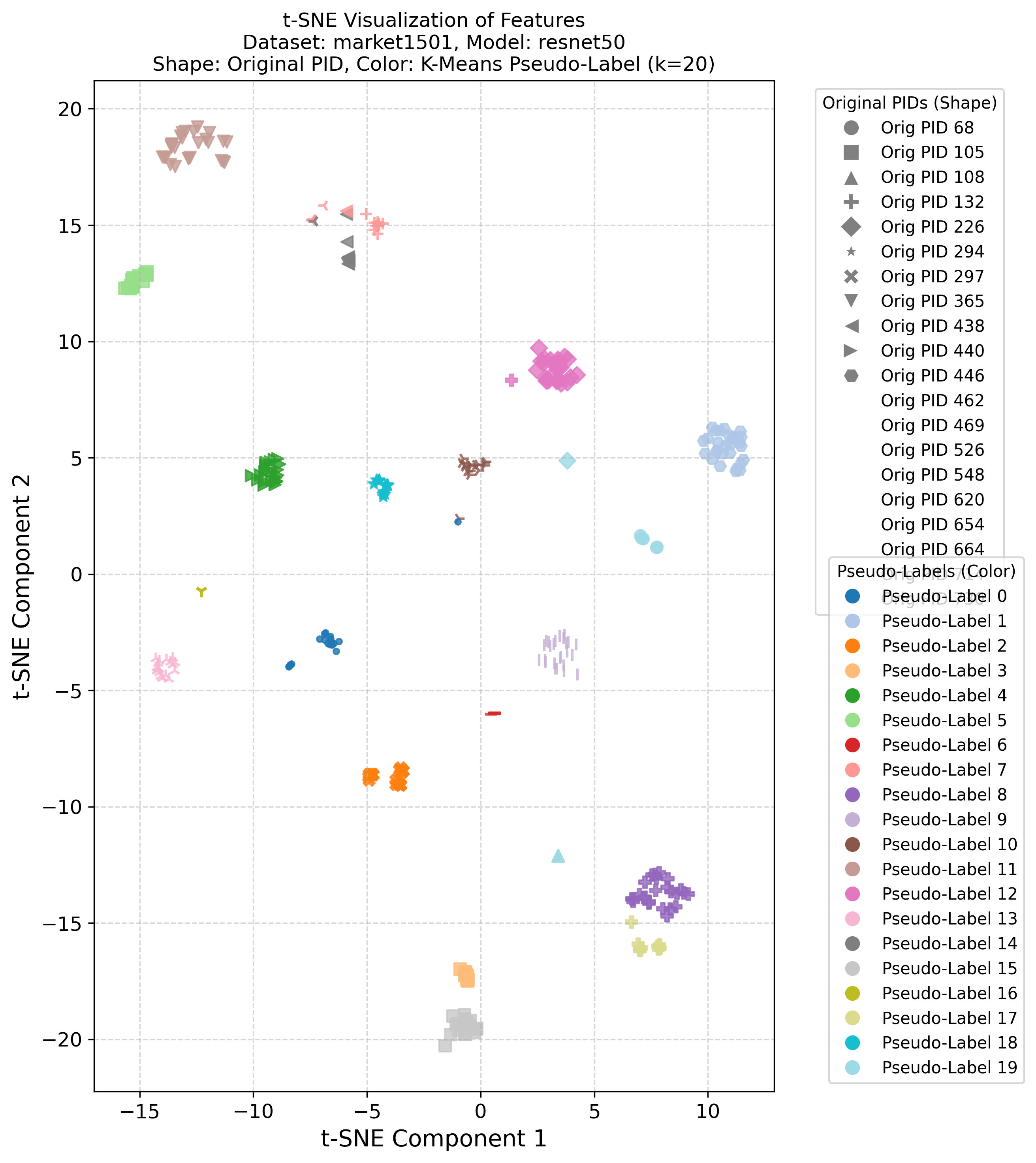

Supplement: S1 Fig — (TIF) [file pone.0328131.s001.tif]

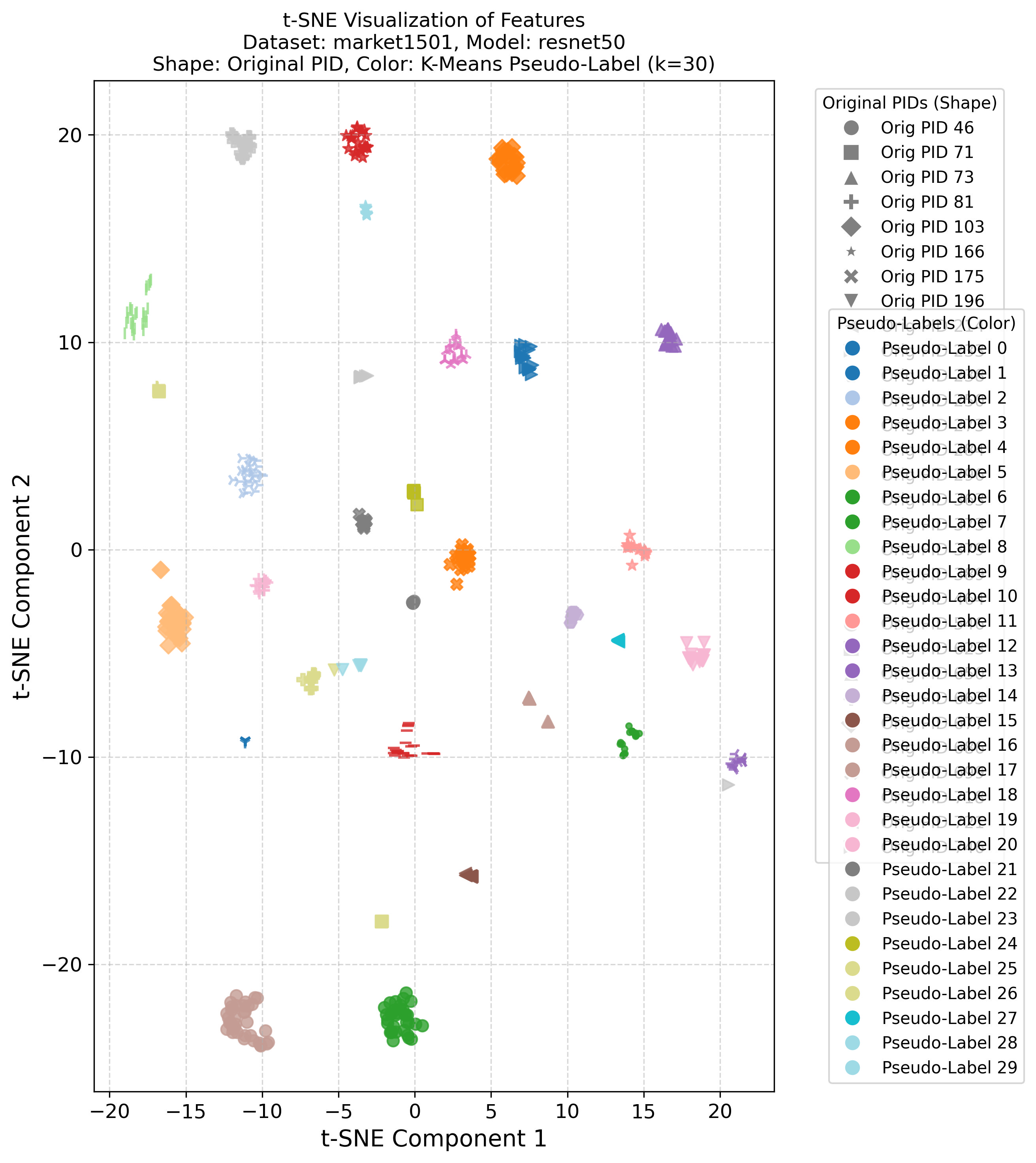

Supplement: S2 Fig — (TIF) [file pone.0328131.s002.tif]

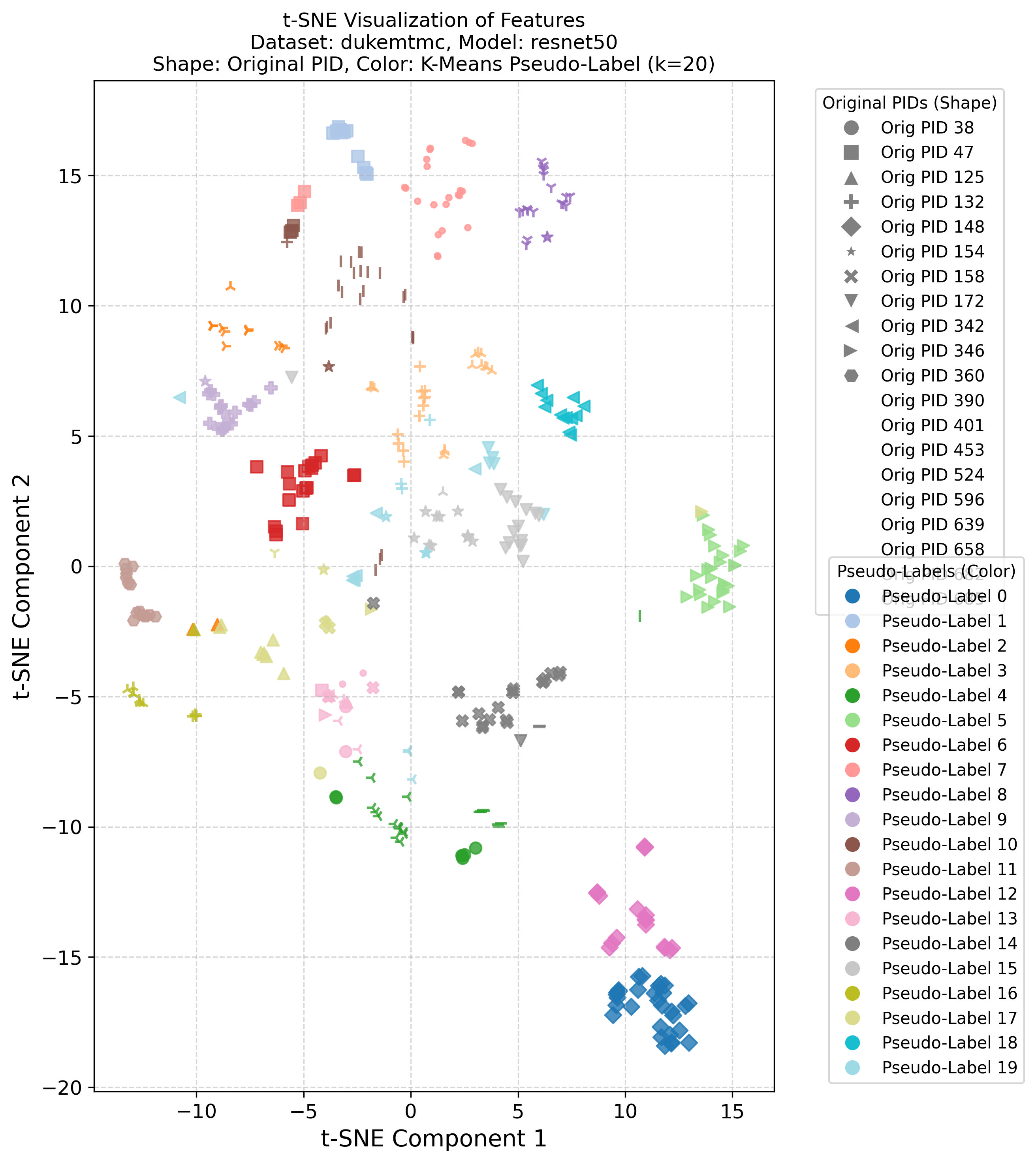

Supplement: S3 Fig — (TIF) [file pone.0328131.s003.tif]

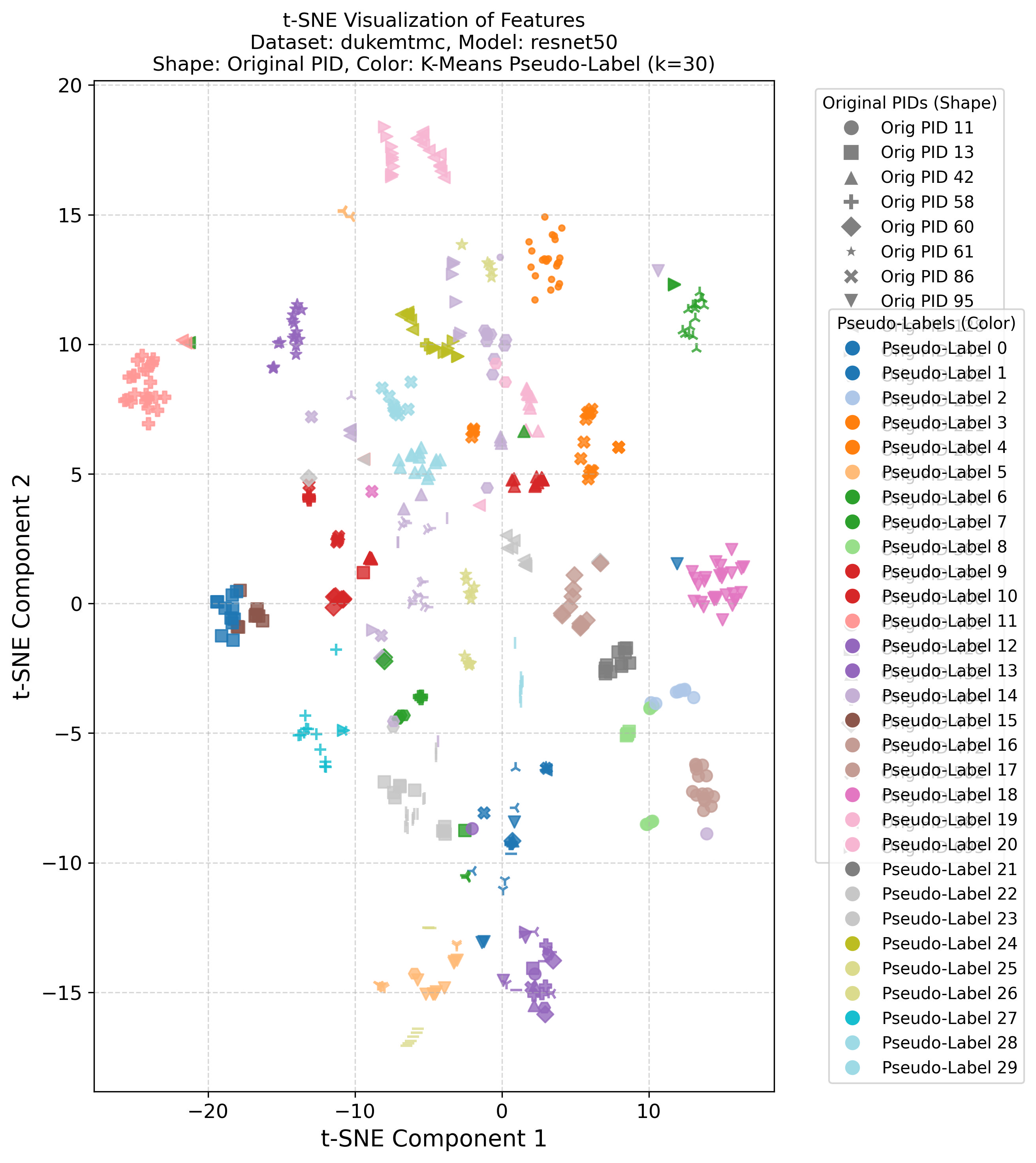

Supplement: S4 Fig — (TIF) [file pone.0328131.s004.tif]

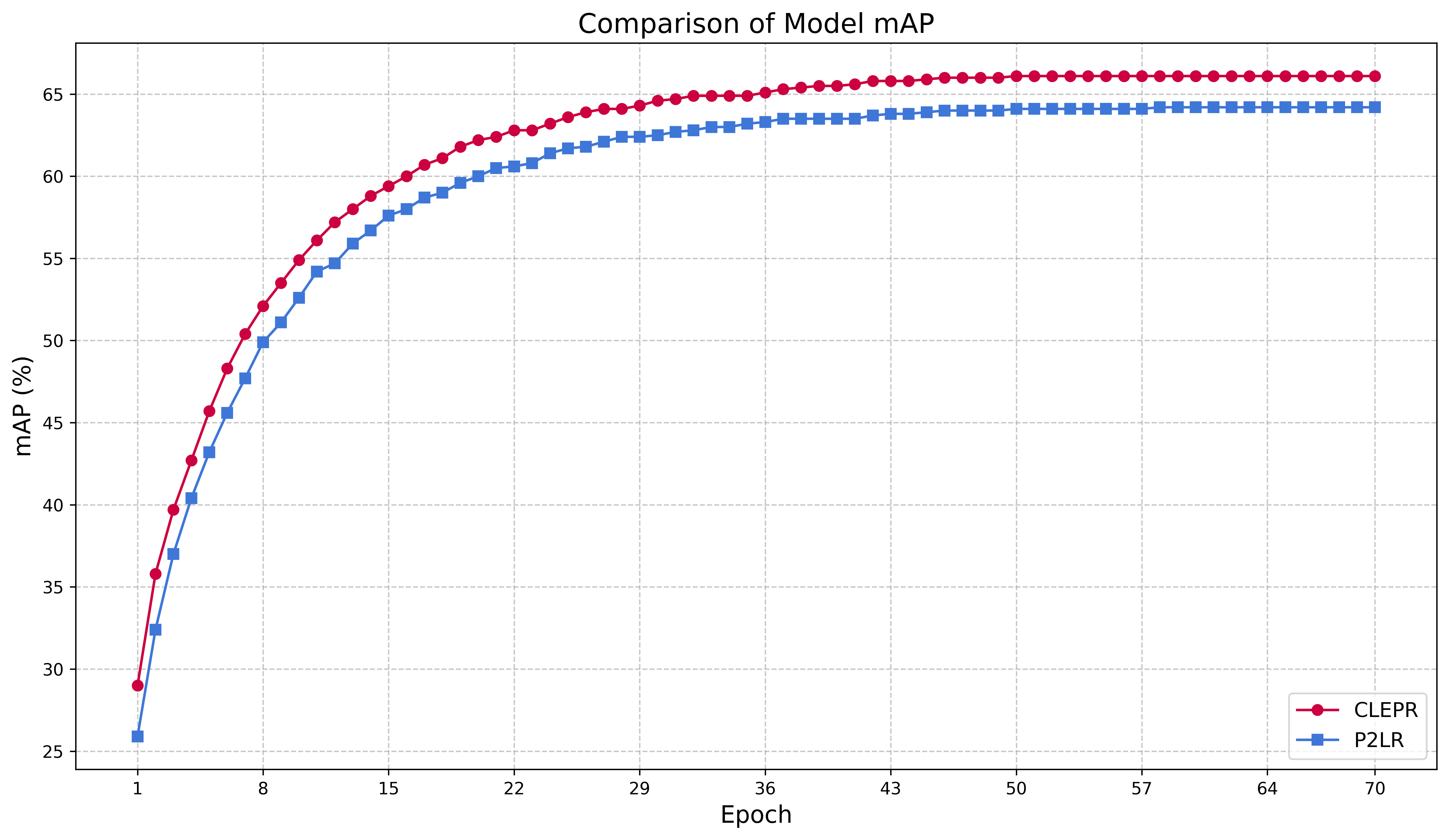

Supplement: S5 Fig — (TIF) [file pone.0328131.s005.tif]

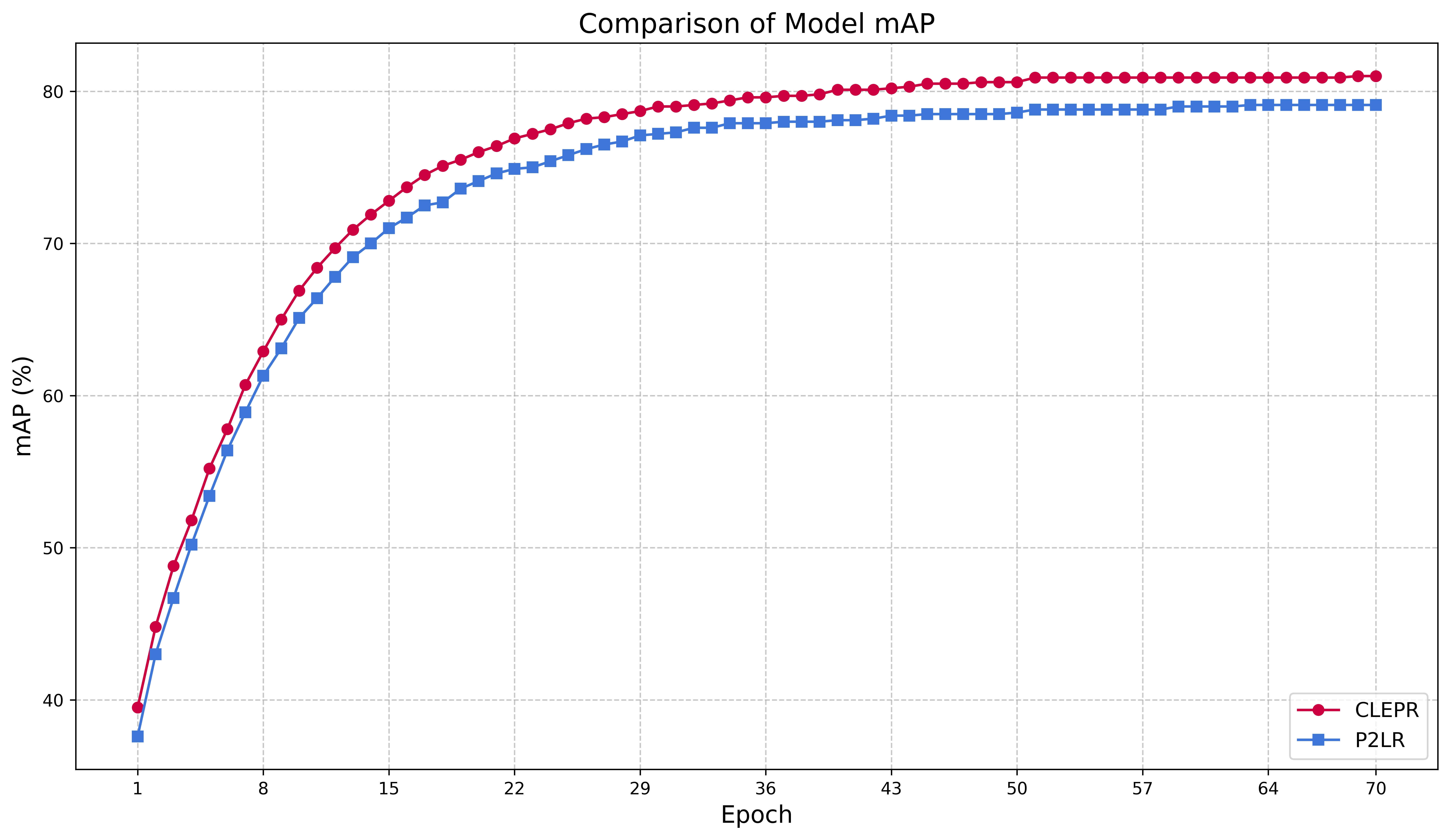

Supplement: S6 Fig — (TIF) [file pone.0328131.s006.tif]

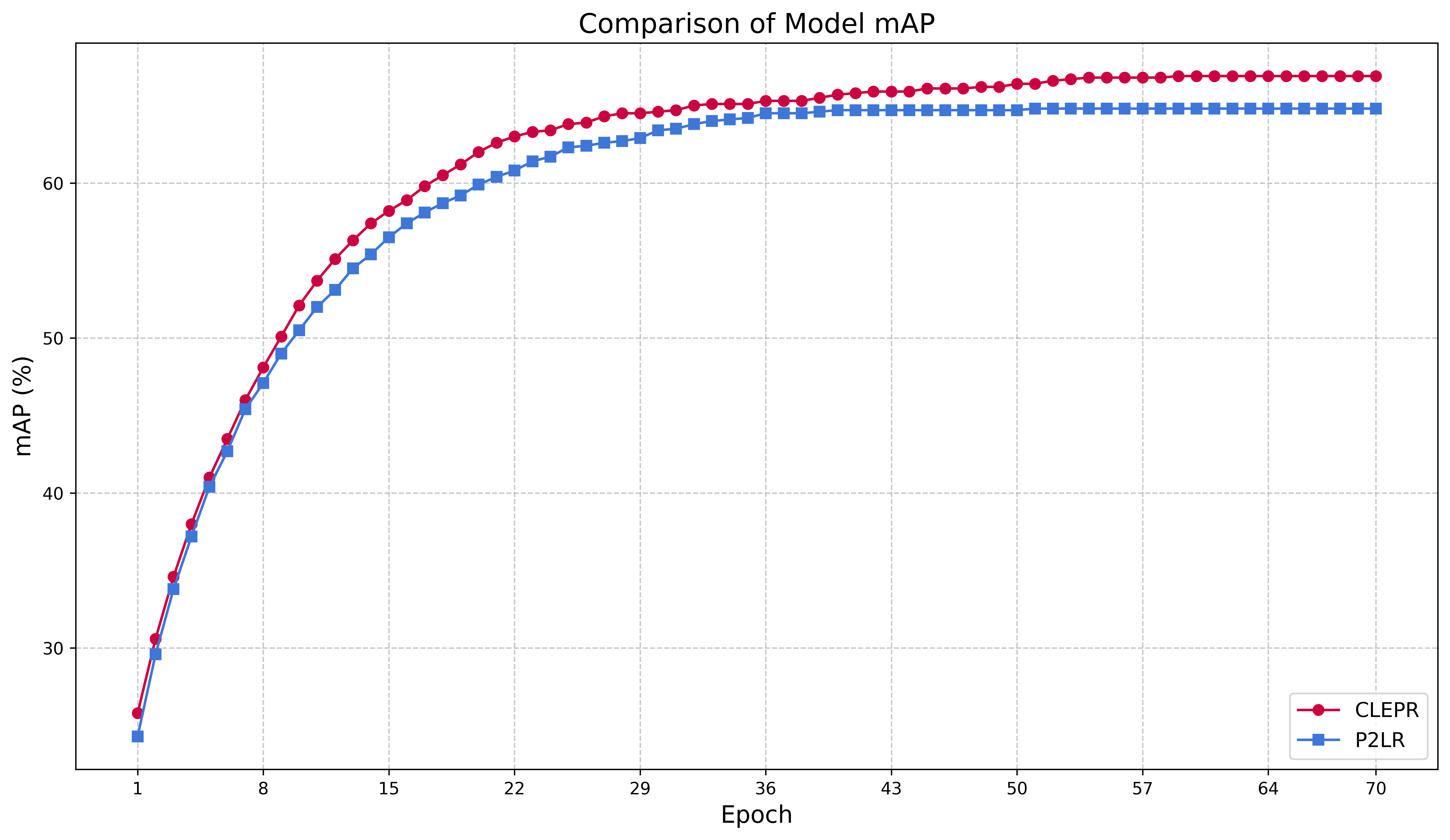

Supplement: S7 Fig — (TIF) [file pone.0328131.s007.tif]

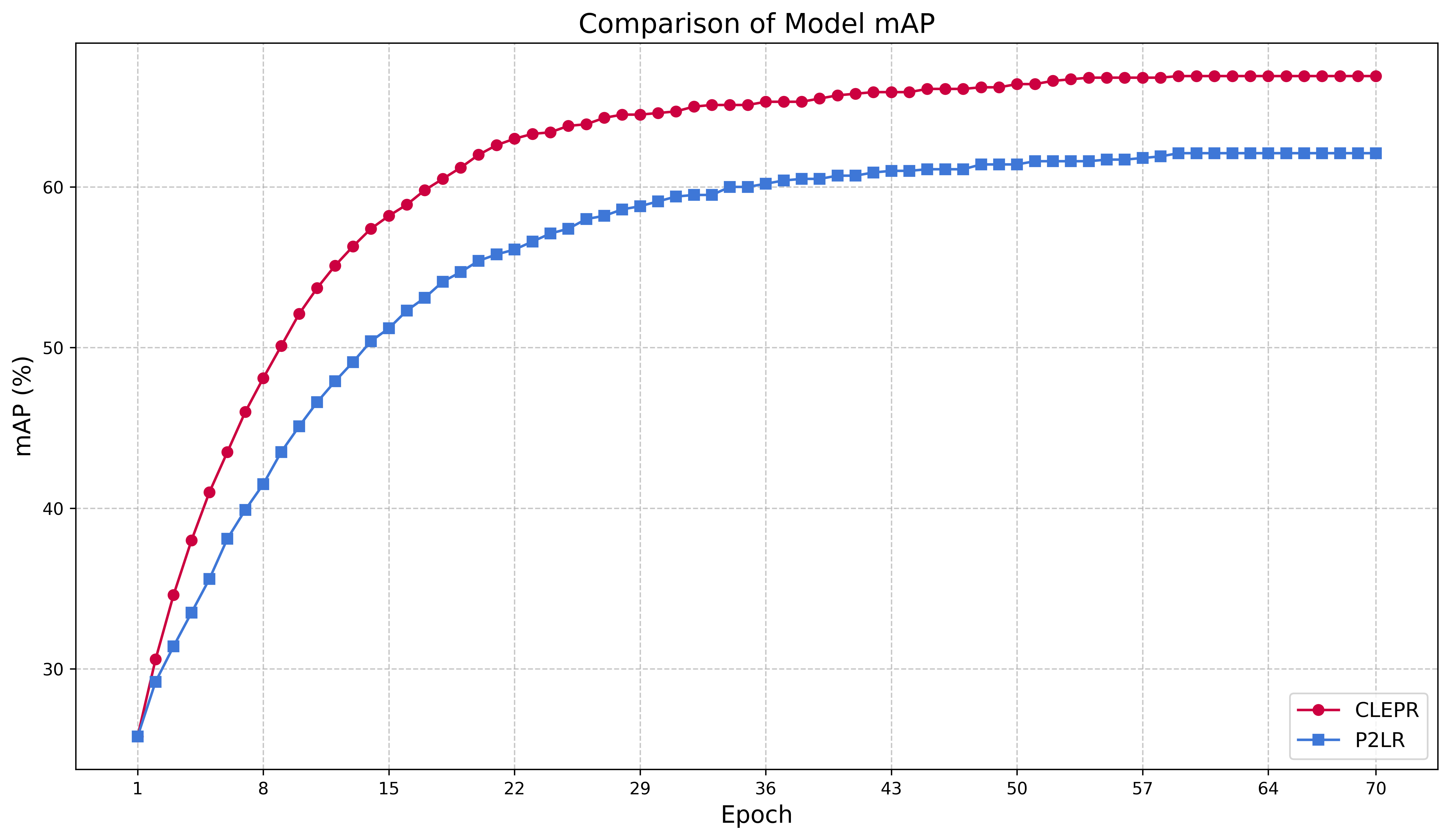

Supplement: S8 Fig — (TIF) [file pone.0328131.s008.tif]

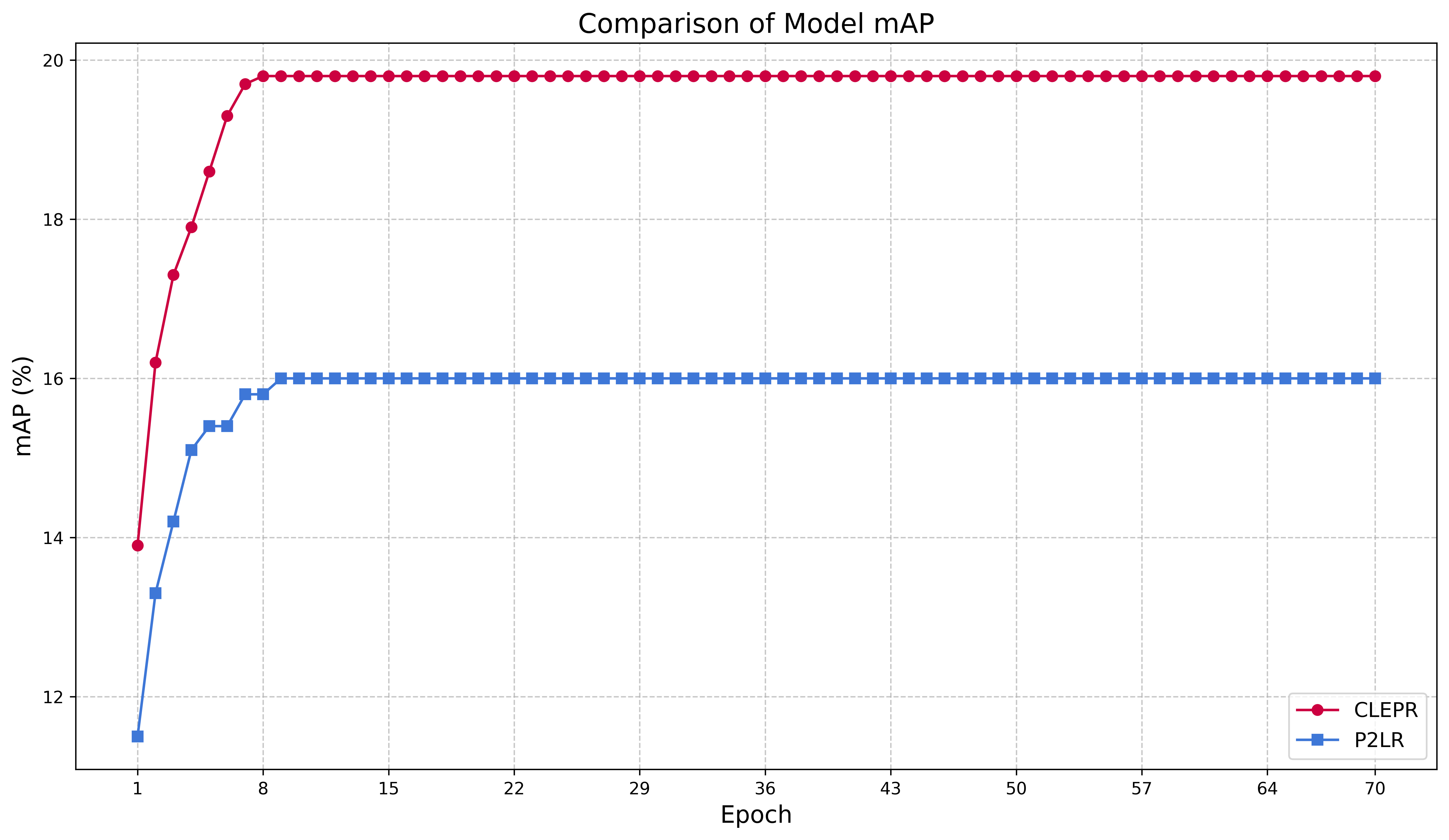

Supplement: S9 Fig — (TIF) [file pone.0328131.s009.tif]
